# TIPS and splenorenal shunt for complications of portal hypertension in chronic hepatosplenic schistosomiasis–A case series and review of the literature

**DOI:** 10.1371/journal.pntd.0010065

**Published:** 2021-12-21

**Authors:** Tamara Nordmann, Stefan Schlabe, Torsten Feldt, Federico Gobbi, Andreas Krieg, Johannes G. Bode, Andre Fuchs, Christian Kraef, Michael Praktiknjo, Jonel Trebicka, Michael Ramharter, Marylyn M. Addo, Christian Strassburg, Ansgar W. Lohse, Tom Luedde, Stefan Schmiedel, Hans Martin Orth

**Affiliations:** 1 Department of Tropical Medicine, Bernhard Nocht Institute for Tropical Medicine & I. Department of Medicine, University Medical Center Hamburg-Eppendorf, Hamburg, Germany; 2 German Center for Infection Research (DZIF), partner site Hamburg-Luebeck-Borstel-Riems, Germany; 3 University Hospital Bonn, Department of Internal Medicine I, Bonn, Germany; 4 German Center for Infection Research (DZIF), partner site Bonn-Cologne, Germany; 5 University Hospital Düsseldorf, Department of Gastroenterology, Hepatology and Infectious Diseases, Düsseldorf, Germany; 6 Department of Infectious/Tropical diseases and Microbiology, IRCSS Sacro Cuore-Don Calabria Hospital, Negrar di Valpolicella, Verona, Italy; 7 Department of Surgery (A), Heinrich-Heine-University and University Hospital Duesseldorf; 8 Internal Medicine III–Gastroenterology and Infectious Diseases, University Hospital of Augsburg, Germany; 9 Centre of Excellence for Health, Immunity and Infections & Department of Infectious Diseases, Rigshospitalet, University of Copenhagen, Denmark; 10 Department of Internal Medicine I, Goethe University Clinic Frankfurt, Germany; 11 Department of Medicine, University Medical Centre Hamburg-Eppendorf, Hamburg, Germany; 12 Department for Clinical Immunology of Infectious Diseases, Bernhard Nocht Institute for Tropical Medicine, Hamburg, Germany; University of Utah, UNITED STATES

## Abstract

**Background:**

Transjugular intrahepatic portosystemic shunt (TIPS) and shunt surgery are established treatment options for portal hypertension, but have not been systematically evaluated in patients with portal hypertension due to hepatosplenic schistosomiasis (HSS), one of the neglected tropical diseases with major impact on morbidity and mortality in endemic areas.

**Methods:**

In this retrospective case study, patients with chronic portal hypertension due to schistosomiasis treated with those therapeutic approaches in four tertiary referral hospitals in Germany and Italy between 2012 and 2020 were included. We have summarized pre-interventional clinical data, indication, technical aspects of the interventions and clinical outcome.

**Findings:**

Overall, 13 patients with confirmed HSS were included. 11 patients received TIPS for primary or secondary prophylaxis of variceal bleeding due to advanced portal hypertension and failure of conservative management. In two patients with contraindications for TIPS or technically unsuccessful TIPS procedure, proximal splenorenal shunt surgery in combination with splenectomy was conducted. During follow-up (mean follow-up 23 months, cumulative follow-up time 31 patient years) no bleeding events were documented. In five patients, moderate and transient episodes of overt hepatic encephalopathy were observed. In one patient each, liver failure, portal vein thrombosis and catheter associated sepsis occurred after TIPS insertion. All complications were well manageable and had favorable outcomes.

**Conclusions:**

TIPS implantation and shunt surgery are safe and effective treatment options for patients with advanced HSS and sequelae of portal hypertension in experienced centers, but require careful patient selection.

## Author summary

Hepatosplenic schistosomiasis is a severe form of chronic infection with various trematodes of the genus *Schistosoma*, characterized by portal venous fibrosis, splenomegaly with hypersplenism, and portal hypertension with subsequent life-threatening bleeding events. While effective anthelminthic treatment is available, portal fibrosis is only partially reversible. Portal hypertension with subsequent bleeding events as a complication is hitherto insufficiently addressed. Surgical techniques are currently the best established treatment option for HSS, despite their inherent complication risk and irreversibility. Interventional procedures like TIPS have rarely been assessed in HSS with mixed results. In a series of 13 cases with follow-up periods up to 99 months and a cumulative follow-up of 30.9 years, we demonstrate excellent bleeding prophylaxis and a low adverse event rate of TIPS and–if TIPS proves infeasible–splenorenal shunt surgery. Main complications of TIPS comprise transient hepatic encephalopathy and increase of liver enzymes, especially in patients with hepatic comorbidities. Due to necessary infrastructure and skills, TIPS implantation is currently limited to specialized centers. However, we think that our study can support the establishment and development of new treatment options for schistosomiasis and, in the medium term, also improve the prognosis of this neglected tropical disease in endemic regions.

## Introduction

Schistosomiasis is a neglected tropical disease caused by infection with parasitic trematodes of the genus *Schistosoma*. Currently, the disease is endemic in 78 countries with more than 250 million people infected and 779 million people at risk worldwide [1,2]. According to the global burden of disease study, in 2019, schistosomiasis was responsible for 11,500 deaths and 1.64 million disability-adjusted life years (DALY) [3]. Older data from 2003 estimated schistosomiasis to cause up to 130,000 deaths annually in Africa by esophageal variceal bleeding [4].

Following percutaneous water-borne infection, adult *S*. *mansoni* (endemic in Africa and parts of the Americas, mainly Brazil), *S*. *intercalatum* (endemic in Africa), *S*. *japonicum* and *S*. *mekongi* (both endemic in South East Asia) worms live in the mesenteric veins and produce up to thousands of eggs per day. These eggs, when released into the portal vascular system, cause chronic inflammation, which in the long term cause a characteristic pattern of periportal fibrosis (pipe-stem or Symmers’ fibrosis) [5–7]. A small percentage of patients (4–8%) develop hepatosplenic schistosomiasis (HSS), characterized by periportal fibrosis with portal hypertension, esophageal varices and splenomegaly with hypersplenism [8].

Since the hepatic parenchyma is not primarily affected, the liver function usually remains stable until advanced disease stages. The underlying causes for developing advanced HSS remain unclear; several factors (parasite burden and egg load, genetic factors, infection in infancy, immunomodulation of T-cell response) and their interplay may be involved in the pathophysiology of HSS [8–11].

Upon treatment with praziquantel, the antiparasitic drug of choice, intestinal symptoms and inflammation improve rapidly, while reversal of hepatic pathology is often delayed and incomplete [12]. Thus, therapy in HSS requires management of portal hypertension to avoid life threatening complications, but the optimal treatment options for advanced disease or secondary prophylaxis after variceal bleeding events are still under discussion. Medical treatment is currently limited to non-selective beta-blockers (NSBB). In case of esophageal varices with and without bleeding, endoscopic management consists of rubber band ligation (RBL) and/or sclerotherapy as in other forms of portal hypertension [13]. Surgical techniques include non-derivative surgery (esophagogastric devascularization with splenectomy (EGDS) / azygoportal disconnection and splenectomy (APDS)) and shunt procedures (distal (DSRS, Warren’s Shunt) and proximal splenorenal shunts), which may also be combined with endoscopic procedures. Currently, surgical procedures are the best researched and most favored definitive therapy for HSS despite their high-grade invasiveness, perioperative mortality and the consequences of post-splenectomy syndrome. [14,15].

Transjugular intrahepatic portosystemic shunt (TIPS) is a minimal invasive procedure and has become a corner stone in the management of portal hypertension and its complications in patients with advanced chronic liver disease [16]. Implementation in HSS, however, is based on limited evidence: One prospective study from 1994 showed unfavorable rates of re-bleeding and hepatic encephalopathy [17], which led to TIPS being discouraged in HSS patients [18]. Since 1994, only few case reports and one small case series of TIPS as treatment for advanced disease have been published [19–21]. Recently, a retrospective study on TIPS in 20 patients with HSS (*S*. *japonicum*) and 62 patients with HBV-induced liver cirrhosis showed comparable results for both groups in terms of hepatic encephalopathy, re-bleeding and survival [22].

Here we present a case series of HSS patients who were treated with TIPS or surgery for primary and secondary prophylaxis of variceal bleeding, after failure of conservative treatment. Four cases had been reported previously with shorter duration of follow-up and with limited data set [19,21].

## Methods

### Ethics statement

Prior to data collection, ethical clearance for publication was obtained from all responsible ethics boards:

1. Ethical Board of the Heinrich-Heine University Düsseldorf, Germany (2020–1225)
2. Ethical Board of the Hamburg Medical Association, Germany (WF-006/21)
3. Ethical Board of the Medical Faculty of the Rheinische Friedrich-Wilhelms-Universität Bonn, Germany (096/16)
4. Ethical Board of the University of Verona. Italy (3079CESC)

The respective ethical boards gave consent for publication of retrospectively collected data without individual consent, since anonymity can sufficiently be granted.

### Study design and inclusion criteria

This case series was compiled from all patients with confirmed HSS treated interventionally or surgically between 2012 and 2020 in four centers in Germany (3), and Italy (1). All examinations and interventions were performed as routine care in accordance with relevant treatment guidelines [23–25]. Data processing was conducted in compliance with good clinical practice and applicable laws. Descriptive analysis was performed using IBM SPSS Statistics for Windows, Version 21 (IBM Corp., Armonk, NY, USA).

### Evaluation of schistosomiasis and liver fibrosis

Diagnosis of schistosomiasis was confirmed by detection of eggs in stool or tissue or positive *Schistosoma spp*. serology in combination with typical history and clinical presentation. Microscopy of merthiolate-iodine-formaldehyde stained stool samples was conducted in all patients, in some cases, unstained rectal biopsies were assessed for presence of live eggs. In two patients, liver biopsies were conducted and stained with hematoxylin and eosin, with both showing *Schistosoma* eggs. Serological diagnostics for *Schistosoma* comprised IIFT, Western Blot, ELISA and ICT with different test methods used in the four centers. Most centers, however, used an IIFT by Euroimmun AG, Lübeck, Germany, with a sensitivity of 92–100% and a specifity of 79–100% [26].

Further evaluation of liver disease included assessment of hepatotropic viruses, gastroscopy for variceal screening, and ultrasonography including measurement of hemodynamic parameters. The hepatosplenic manifestation was determined by typical sonographical findings, collateral circulation with gastric, esophageal or rectal varices, and a low platelet count as a sign of hypersplenism. Ultrasound protocols were not consistent between centers. However, the following parameters were documented in all examinations: Splenic length, dorso-ventral and cranio-caudal liver size in the midclavicular line, portal venous flow, and descriptive assessment of liver texture, vascularization, and surface area, as well as a non-graded description of periportal fibrosis. For grading of esophageal varices, Paquet’s classification was applied in all centers.

### TIPS

TIPS was considered in patients with recent or uncontrolled variceal bleeding (emergency or preemptive TIPS). Additionally, TIPS was considered for primary or secondary prophylaxis, if regression or stabilization of varices was not achieved using NSBB or RBL. For assessment of vascular structure and cardiac function a contrast-enhanced CT scan of the abdomen and an echocardiography were performed. Moreover, hepatic encephalopathy (West-Haven-criteria, flicker frequency measurement, number-connection test) was assessed preinterventionally and during follow-up appointments.

The technique of TIPS implantation is well established at the respective medical centers. After transjugular catheterization of the right hepatic vein and ultrasound- or CT-guided transhepatic puncture of an intrahepatic branch of the portal vein, a portosystemic stent was inserted and dilated to the desired diameter. Successful placement and shunting were confirmed by angiography. Intravascular pressures of caval and portal veins were measured invasively during the procedure before and after placing the stent. The goal of the procedure was a decrease of >50% of the portosystemic pressure gradient (PSPG), preferably below 12 mmHg.

Postinterventional inpatient care was continued for about one week. All patients received therapeutic PTT-guided anticoagulative therapy for 24h followed by prophylactic anticoagulation with low molecular weight heparin for another week. Additional platelet aggregation inhibitors (e.g. clopidogrel) was administered in some patients (e.g. with uncovered stents). Lactulose was administered routinely for prevention of hepatic encephalopathy.

### Shunt surgery

For patients in whom TIPS implantation was contraindicated or technically not successful, surgery was conducted according to established technique: Following upper abdominal laparotomy and exposure of the spleen, the feeding vessels are severed and the spleen is removed; the splenic vein is dissected along the upper edge of the pancreas up to the portal venous confluence and anastomosed laterally to the likewise dissected left renal vein.

### Follow-up

At quarterly follow-up appointments detailed history was taken, focusing on signs of hepatic and cardiac decompensation and bleeding episodes. Examinations included liver, kidney and coagulation function tests, a complete blood count, a structured hepatic encephalopathy assessment, abdominal ultrasound and duplex sonography. Follow-up gastroscopy was usually conducted three months after interventions and later as recommended by the treating physician. Despite not being evaluated for severity of hepatic schistosomiasis, Child-Pugh and Model of End-stage Liver Disease (MELD) scores were calculated to monitor pre- and postinterventional liver function.

## Results

### Patient characteristics, therapy and outcome after diagnosis of schistosomiasis

We included 13 patients (77% male) aged 16 to 59 years (median 27 years) (Table 1). Although species identification was not available in all cases, it can be assumed that 12 patients with origin in sub-Saharan Africa were infected with *S*. *mansoni* and one patient, who had migrated from the Philippines, with *S*. *japonicum*. In all cases antiparasitic treatment with praziquantel was administered.

**Table 1:** Patients characteristics.

Pat. No.	Sex	Country of origin	Schistosoma species†	Year of praziquantel treatment	Year of initial presentation	Age at initial presentation (years)	Latest follow-up	Year of TIPS / shunt surgery	Follow-up period since TIPS / shunt surgery (months)	Medical history
1	m	Guinea	*S*. *mansoni*	2011 and 2012	2012	19	2020	2012	99	interventional ASD-occlusion, sickle cell trait
2	m	Zaire	*S*. *mansoni*	2002	2014	39	2020	2016	45	none
3	m	Eritrea	*S*. *mansoni*	2014	2014	28	2015	2014	12	regular alcohol consumption, iron deficiency
4	f	Madagascar	*S*. *mansoni*	2015	2015	37	2018	2015	27	partial portal venous thrombosis
5	m	Eritrea	*S*. *mansoni*	2016	2016	19	2017	2016	8	chronic hepatitis B
6	m	Sierra Leone	*S*. *mansoni*	2016	2016	16	2020	2016	48	none
7	m	Eritrea	*S*. *mansoni*	2016	2017	20	2019	2017	30	*H*.*pylori* gastritis, esophageal candidiasis
8	m	Eritrea	*S*. *mansoni*	2017	2017	27	2020	2019	8	none
9	f	Philippines	*S*. *japonicum*	2017	2017	59	2020	2017	34	DM2, inactive hepatitis B, HTN, iron deficiency
10	m	Eritrea	*S*. *mansoni*	2018	2018	38	2020	2018	23	antral gastritis
11	m	Guinea	*S*. *mansoni*	2018	2018	21	2020	2018	20	antral gastritis
12	m	Eritrea	*S*. *mansoni*	2018	2018	18	2018	2018	0	portal venous thrombosis
13	f	Eritrea	*S*. *mansoni*	2018	2018	35	2020	2018	17	recurrent cholangitis due to chronic fascioliasis, SBP, portal venous thrombosis

Abbreviations: TIPS = transjugular intrahepatic portosystemic stent shunt; ASD = atrial septal defect; SBP = spontaneous bacterial peritonitis; DM2 = diabetes mellitus type 2; HTN = arterial hypertension; † microscopically confirmed or assumed based on patient’s geographic origin

Previous bleeding events were documented in 9 cases (70%). Ten patients (77%) underwent RBL of esophageal varices, one patient (8%) was additionally treated with endoscopic histoacryl injection (Table 2). Upon confirmation of varices without signs of imminent bleeding or history of gastrointestinal bleeding, usually NSBB was started.

**Table 2:** HSS characteristics and treatment (portal hypertension, splenomegaly, collateral circulation) of all patients.

Pat. No.	Spleen length (mm)	Esophageal varicosis (pre-intervention), Degrees Paquet’s classification	NSBB	AE under NSBB therapy	Bleeding events	Interventions	TIPS / shunt surgery
1	260	II following RBL	Dosage not documented	hypotension	more than one event	RBL	2012, 2x revision
2	168	III	Carvedilol 6.25mg bid	hypotension	none	RBL	2016
3	265	III + red spots	none	NA	none	RBL	2014
4	130	III	Propranolol 20mg bid	discontinued after one month (hypotension)	hematemesis	RBL	2015
5	297	II + red spots	Propranolol 10 mg bid	hypotension	esophageal variceal bleeding	RBL	2016
6	210	III + red spots	none	NA	hematemesis (min 2x) + hematochezia	RBL	2016
7	188	III + red spots	Propranolol 10mg tid	none documented	hematochezia	none	2017
8	212	II + red spots, 2 GE varices	Carvedilol 12.5mg bid	none documented	none	RBL	2019
9	132	II/III	Carvedilol 12.5mg bid	discontinued after 1 month	hematemesis, esophageal variceal bleeding (min. 3x)	RBL	2017
10	211	II; Rectal varicosis w. red spots	none, due to low baseline blood pressure	NA	hematochezia from rectal varicosis	none	shunt surgery 2018
11	177	III + red spots, GE varices Type I, Fundus varices	Carvedilol 3.125mg bid	none documented	none documented	none	2018, 2x revision
12	192	III + red spots, 2 GE varices Type I	Propranolol 10mg bid	none documented	hematochezia	RBL	2018
13	213	III + red spots	Carvedilol 6.25mg bid	none documented	hematemesis	RBL + histoacryl	shunt surgery 2018

Abbreviations: AE = adverse events; RBL = rubber band ligation; bid = bis in die (two per day); tid = ter in die (three per day); TIPS = transjugular intrahepatic portosystemic shunt

### Indication and performance of TIPS insertion

In twelve patients (93%) indication for TIPS insertion was established, details on indication and technical procedure are shown in Table 3. These patients had higher-grade varices (II° - IV°, 11/12, 92%), signs of imminent bleeding (red spots, 8/12, 67%) or previous bleeding events (8/12, 67%). Three patients (25%) had more than one episode of variceal bleeding and 9/12 (75%) had previously undergone RBL.

**Table 3:** Outcome of TIPS insertion.

		Before TIPS, earliest available values					TIPS Procedure						After TIPS, most recent values						
Pat. No.	Follow-up period since TIPS (months)	Spleen length (mm)	Child-Pugh	MELD	Esophageal varicosis (pre-intervention), (degrees Paquet’s classification)	Initial platelet count (nl^-1^)	Stent type	PSPG before (mmHg)	PSPG after (mmHg)	Delta (mmHg)	Delta (%)	Spleen length (mm)	HE (grade)	Child-Pugh	MELD	Esophageal varicosis (degrees Paquet’s classification)	Reduction (degrees Paquet’s classification)	Latest platelet count (nl^-1^)	Complications following TIPS
1	99	260	5 (A)	11	II following RBL	54	Bard Luminexx	23	12	11	47.8	240	none	7 (B)	15	I	0	58	2x stent thrombosis, HE
2	45	168	5 (A)	9	III	37	Gore Viatorr	23	6	17	73.9	140	none	7 (B)	10	I-II	1–2	41	none
3	12	265	5 (A)	5	III + red spots	50	Bard E-Luminexx + Fluency	13	1	12	92.3	..	none	5–7 (A/B) *	11	none	3	40	none
4	27	130	6 (A)	7	III	144	Gore Viatorr	..	16	NA	NA	100	none	6 (A)	8	none	3	227	none
5	8	297	6 (A)	15	II + red spots	23	Gore Viatorr	19	9	10	52.6	172	I-II	11 (C)	28	I	1	21	Jaundice, HE
6	48	210	5 (A)	10	III + red spots	20	Bard E-Luminexx+ Fluency	23	11	12	52.2	280	none	5 (A)	12	none	3	..	none
7	30	188	5 (A)	8	III + red spots	52	Gore Viatorr	50	27	23	46.0	151	none	7 (B)	16	I	2	64	HE
8	8	212	5 (A)	10	II + red spots, 2 GE varices	68	Gore Viatorr	15	7	8	53.3	210	none	6 (A)	15	I	2–3	56	none
9	34	132	6 (A)	13	II/III	103	Bard E-Luminexx + Fluency	24	8	16	66.7	130	I	8 (B)	12	none	2–3	69	impaired liver function, HE
11	20	177	6 (A)	14	III + red spots, GE varices Type I, Fundus varices	65	Gore Viatorr, Boston Scientific EPIC (2nd Revision)	14	8	6	42.9	150	none	8 (B)	18	I-II	1–2	71	2x stent thrombosis; elevated liver enzymes
12	0	192	5 (A)	9	III + red spots, 2 GE varices Type I	57	Gore Viatorr	25	13	12	48.0	166	I	8 (B)	13	I	2	70	catheter associated sepsis, portal vein thrombosis, HE

Abbreviations: TIPS = transjugular intrahepatic portosystemic shunt; MELD = model for end-stage liver disease; PSPG = portosystemic pressure gradient; HE = hepatic encephalopathy; GE = gastroesophageal; NA = not applicable

* = underlying data incomplete; Child-Pugh score: 5–6: A, 7–9: B, 10–15: C

In ten patients TIPS insertion was successful at the first attempt (technical success rate 83%) despite significant stiffness of the fibrotic periportal tissue. In one of the two cases with unsuccessful first attempt of TIPS insertion, a second attempt was successful under general anesthesia. The other case underwent shunt surgery after two frustrating attempts of TIPS insertion: A first attempt failed due to poor sonographic visibility, in a second attempt the portal vein could not be punctured due to the anatomical changes.

In 10/11 patients (91%) hemodynamic values of TIPS procedure were available. A reduction of the PSPG below 12 mmHg or below 50% of baseline was achieved in 7/10 patients (70%).

No major periinterventional complications (e.g. major bleeding, hepatic laceration) occurred and no post-TIPS cardiac decompensation was observed. One patient developed central venous catheter associated sepsis with hepatic encephalopathy and partial portal venous thrombosis which were managed without further complications.

Following TIPS insertion, a major deterioration of liver function with an increase of MELD score up to 28 was observed in one case, presumably aggravated by concomitant chronic hepatitis B virus infection. The patient later underwent liver transplantation at another center. In another patient with suspected liver cirrhosis due to diabetes mellitus type 2 and inactive hepatitis B virus infection, a significant increase in liver enzymes was observed. Both patients showed de-novo episodes of hepatic encephalopathy which subsided under intensified treatment with oral lactulose and/or rifaximin. In the majority of patients, a mild increase of Child-Pugh and MELD scores was noticeable, which was mainly caused by elevated bilirubin levels. Table 4 shows the underlying data for Child-Pugh and MELD scores in these patients.

**Table 4:** Detailed evaluation of Child-Pugh and MELD score following TIPS.

	Before TIPS, earliest available values								After TIPS, most recent values							
Pat. No.	Bilirubin (mg/dl)	INR	Creatinine (mg/dl)	Albumin (g/dl)	Ascites (absent / mild / moderate+)	HE (grade)	Child-Pugh	MELD	Bilirubin (mg/dl)	Creatinine (mg/dl)	INR	Albumin (g/dl)	Ascites (absent / mild / moderate+)	HE (grade)	Child-Pugh	MELD
1	1,02	1,5	0,7	4,2	absent	0	5 (A)	11	3,45	0,64	1,4	4,2	absent	0	7 (B)	15
2	1,35	1,1	0,8	3,8	absent	0	5 (A)	9	1,49	0,88	1,2	2	absent	0	7 (B)	10
3	0,3	1,27	0,62	4,6	absent	0	5 (A)	5	0,7	0,64	1,5	..	absent	0	5–7 (A/B)*	11
4	0,49	1,09	0,62	3,28	absent	0	6 (A)	7	0,49	0,62	1,2	3,31	absent	0	6 (A)	8
5	2,22	1,6	0,5	3,8	absent	0	6 (A)	15	21,91	0,4	2,4	2	absent	I-II°	11 (C)	28
6	0,5	1,4	1	3,5	absent	0	5 (A)	10	1,5	1	1,4	3,5	absent	0	5 (A)	12
7	0,74	1,2	0,61	4,74	absent	0	5 (A)	8	3,22	0,6	1,6	3,77	absent	0	7 (B)	16
8	1	1,4	0,9	4,2	absent	0	5 (A)	10	2,7	0,79	1,5	4,3	absent	0	6 (A)	15
9	2,8	1,3	0,77	6,99	absent	0	6 (A)	13	2,7	0,8	1,2	2,9	absent	I°	8 (B)	12
11	2,51	1,3	1,1	4,6	absent	0	6 (A)	14	4,09	0,84	1,7	3,8	absent	0	8 (B)	18
12	1,24	1,2	0,54	4,43	absent	0	5 (A)	9	3,07	0,46	1,2	3,69	absent	I°	8 (B)	13

Abbreviations: TIPS = transjugular intrahepatic portosystemic shunt; MELD = model for end-stage liver disease; HE = hepatic encephalopathy

* = underlying data incomplete; Child-Pugh score: 5–6: A, 7–9: B, 10–15: C

### Shunt surgery

Shunt surgery was conducted in the patient with unsuccessful TIPS insertion and another patient with portal venous thrombosis. Decision was made for proximal splenorenal shunt surgery in combination with splenectomy to address both portal hypertension and hypersplenism. Both patients developed a transient thrombocytosis (max. 967/nl and 831/nl) (Table 5), which may have contributed to the development of an irreversible shunt occlusion in one patient despite anticoagulative therapy with rivaroxaban in combination with ASA for the inhibition of platelet aggregation. However, even in this patient, follow-up revealed complete regression of esophageal varicosis (previously III°). In the other patient, dabigatran was administered for a total of 12 months and the shunt remained patent during the follow-up time of 23 months without major bleeding events.

**Table 5:** Outcome of shunt surgery.

		Before shunt, earliest available value					Surgery	After shunt, most recent values						
Pat. No.	Follow-up period since surgery (months)	Spleen length (mm)	Child-Pugh	MELD	Initial platelet count (nl^-1^)	Esophageal varicosis (pre-intervention), (degrees Paquet’s classification)	Surgery conducted	HE (grade)	Child-Pugh	MELD	Esophageal varicosis (degrees Paquet’s classification)	Reduction (degrees Paquet’s classification)	Latest platelet count (nl^-1^)	Complications following surgery
10	23	211	5 (A)	12	43	II; rectal varicosis w. red spots	Proximal splenorenal shunt, splenectomy	none	5 (A)	11	none, rectal varicosis regredient	2	200	Transient thrombocytosis
13	17	213	5 (A)	9	53	III + red spots, GE varices	Proximal splenorenal shunt, splenectomy, cholecystectomy, partial pancreatectomy, removal of splenic artery aneurysm	none	5 (A)	7	I	2	518	Thrombocytosis, shunt and portal vein thrombosis despite oral anticoagulation

Abbreviations: TIPS = transjugular intrahepatic portosystemic shunt; MELD = model for end-stage liver disease; HE = hepatic encephalopathy; GE = gastroesophageal; Child-Pugh score: 5–6: A, 7–9: B, 10–15: C

### Follow-up

The median follow-up period after TIPS or shunt surgery was 23 months (0–99 months), with a cumulative follow-up time of 30.9 patient years. No variceal bleeding events were seen during this time.

After TIPS or shunt surgery, esophageal varices were distinctively reduced with a median reduction of 2 degrees according to Paquet’s classification. In six patients (46%), no varices were detectable in the most recent gastroscopy. In five of the remaining seven patients, varices were grade I, in two patients, grade I-II. No risk patterns for imminent variceal bleeding were observed.

Rectal varicosis, as indication for shunt surgery in one patient, also showed regression in follow-up.

Stent thrombosis was observed twice in two patients each. In both cases, re-interventions and the addition of ASA or apixaban, resulted in sustained stent patency without bleeding complications (further follow-up: 49 and 10 months, respectively).

In addition to the three previously mentioned patients with hepatic encephalopathy immediately after TIPS insertion, two more patients experienced de-novo episodes of overt hepatic encephalopathy during later follow-up. One of these episodes was associated with a gastrointestinal infection, the other with discontinuation of prophylactic lactulose treatment. Both subsided upon increasing or restart of the lactulose therapy and addition of rifaximin.

## Discussion

This case study provides evidence that TIPS and shunt surgery are safe, effective, and sustainable treatment options in carefully selected patients and at experienced treatment centers. To the best of our knowledge, this study contains the largest patient group with interventional therapy of HSS due to *S*. *mansoni* in the setting of high-income countries. We were able to assess a comprehensive set of parameters before and after the respective interventions during the follow-up period of 30.9 patient years.

NSBB therapy has been shown to improve hemodynamic parameters and to reduce mortality in patients with HSS [27,28]. In 11 of our patients NSBB treatment was initiated, but in four patients (36%) the dose had to be reduced or discontinued due to arterial hypotension and bradycardia. In one patient each, treatment was discontinued without documented reasons or could not be initiated due to preexisting hypotension. A possible explanation for this high rate of intolerance to NSBB therapy may be the relatively young median age of 27 years with usually lower systemic blood pressure in contrary to 34.6 and 40 years, respectively, in the above-mentioned studies [27,28].

Although TIPS is well established for the therapy of various complications of portal hypertension, only very few reports exist for the use in HSS [17–21], illustrating the lack of treatment options for sequelae of this neglected disease. Comparison of our data with the previous studies from Egypt and China is problematic, considering the limited resources of the respective health care settings and the different disease characteristics. The Egyptian study included a high rate of coinfections with hepatitis B and C virus infection (26% and 71%, respectively) and the Chinese study–given the local epidemiology–presumably deals with *S*. *japonicum*, while 91% of our patients undergoing TIPS implantation were infected with *S*. *mansoni*. Moreover, 12 of the 20 Chinese patients had a history of splenectomy before TIPS insertion, which may explain altered portal hemodynamics.

In eleven of our patients, TIPS could be successfully implanted with satisfactory hemodynamic results (PSPG < 12mmHg or <50% of baseline) in 7/10 patients with available documentation. These results are comparable to those of the Egyptian and the Chinese studies (PSPG < 12 mmHg in 66% and 90%, respectively) and are in line with the pre-sinusoidal nature of disease. One important finding was the distinctive regression of varices in our patients (2 degrees median reduction). During follow-up, no variceal bleeding episodes were observed. In this small number of patients, complete prevention of re-bleeding was achieved while episodes of re-bleeding were seen in the Egyptian and the Chinese study in up to 25% during mean follow-up times of 24 and 15 months, respectively.

Here, a possible positive effect of using more advanced TIPS technology must be considered. In the Egyptian study in 1994 only uncovered stents were implanted: Memotherm (25), Wallstent (13) and Symphony (6). In patients with liver cirrhosis, improved long term patency could be shown for newer polytetrafluoroethylene covered stents [29], which were used in ten of our 11 patients (91%). During follow-up, 2/11 patients (18%) experienced stent thrombosis, one of whom had been treated with an uncovered stent. The other patient had discontinued clopidogrel treatment. In both cases, re-interventions led to sustained stent patency. The findings of the Egyptian study underline, that stent patency is crucial for bleeding prophylaxis since re-bleeding episodes were seen only in patients with stent shunt malfunction.

The rates of hepatic encephalopathy in HSS patients after TIPS implantation in the Egyptian study and the Chinese study were reported with 19% and 25% respectively, comparable to post-interventional results of patients with hepatitis B-associated liver cirrhosis [22]. In our case series, we observed encephalopathy episodes which required hospitalization in five patients (39%), two of whom had preexisting liver dysfunction due to hepatitis B-coinfection. In two patients, the episode of encephalopathy was observed during an acute infection.

Liver function in patients with HSS is generally unimpaired, with isolated portal hypertension due to periportal fibrosis. Hepatic encephalopathy episodes are usually not observed in HSS patients without concomitant liver disease but after TIPS insertion, encephalopathy seems to be associated with the degree of portosystemic shunting. Insertion of smaller diameter TIPS stents or underdilation of TIPS stents may be considered to reduce hepatic encephalopathy risk in HSS patients [30]. However, after the administration of lactulose and rifaximin or management of the underlying infection, no episodes of encephalopathy reoccurred. The favorable outcomes may be attributed to the good hepatic reserve and supporting medical treatment.

The noticeable elevation of Child-Pugh and especially MELD scores following TIPS was mostly attributable to elevated bilirubin levels, with predominantly unconjugated bilirubin indicating hemolysis. The previously mentioned studies from China and Egypt do not contain information on MELD score before and after TIPS-implantation in schistosomiasis patients. While persistently, but more markedly elevated bilirubin levels have been associated with increased mortality following TIPS in liver cirrhosis, all of our patients have been alive throughout the entire follow-up period [31]. As in other intravascular devices, a variable degree of mechanical hemolysis has been described following TIPS implantation in patients with liver cirrhosis. In those patients, hemolysis is mostly transient and appears to be less frequent than in our patients [32]. However, hemolysis is less likely in newer covered TIPS [33], so the bilirubinemia may most likely be attributable to a combination of reduced liver perfusion via shunting and hemolysis owing to hypersplenism. Portal hyperflow in our HSS patients with massively enlarged spleens may contribute to the higher degree of hemolysis. Currently, no clinical significance is apparent from the bilirubin increase, but future studies should keep this finding in mind.

The periinterventional mortality in the Egyptian study was 2%, one patient died due to cardiac tamponade. Moreover, the authors experienced technical difficulties caused by the stiffness of the periportal tissue in line with our observations. The 30-days-mortality of the Egyptian study was 14%. Most deceased patients had received the stent for ascites control and in this group with poor hepatic reserve, the complication rate due to stent thrombosis was higher. The 12-months-mortality in the Chinese study was 15%, the causes of death were variceal re-bleeding in two cases and renal failure in one case. Interestingly, in this study a high rate of ascites was recorded pre- and postinterventionally. In contrary, in our TIPS cohort with mainly *S*. *mansoni* (10/11, 91%) no ascites was documented, probably due to a slightly different pathomechanism in *S*. *japonicum* [34].

Important complications after TIPS insertion in our case series comprised jaundice in a patient with concomitant chronic hepatitis B and deterioration of liver function in another patient suffering from diabetes mellitus type 2 and inactive hepatitis B. Both patients remained in stable condition throughout the follow-up period (8 and 37 months, respectively).

Altogether, the outcome in terms of severe complications, re-bleeding and mortality was favorable in our small cohort of patients with HSS after TIPS or shunt surgery. Surgical techniques, however, still have their value in patients with contraindication for TIPS or unsuccessful TIPS insertion.

Moreover, a remarkable improvement in platelet count was observed following splenectomy. Transient thrombocytosis may have been the reason for shunt occlusion in one of the patients despite therapeutic anticoagulation. Nevertheless, this patient showed complete resolution of esophageal varicosis due to the absence of splenic hyperflow.

A previous study demonstrated small improvement in platelet count after spleen-preserving portosystemic shunt surgery [35]. However, we observed persistent thrombocytopenia (61±34/nl vs. 72±54/nl) after TIPS insertion. Although no bleeding complications were documented during the follow-up period, persistent thrombocytopenia remains a potential risk for bleeding complications. Incomplete splenectomy or splenic autotransplantation may be future options to address the bleeding risk without immunological sequelae of splenectomy [36].

For the consideration of a possible introduction of the TIPS procedure in endemic regions, cost efficiency is one of the decisive criteria. Recent studies from Europe or North America have assessed the cost of TIPS procedure in patients with liver cirrhosis, which ranged from £4,646 (currently €5,509) [37] to $50,865.94 (currently €43,692) [38]. Since these calculations do not itemize personnel costs, consumables, profit margins, or basic technical equipment, a transfer to the economic situation in endemic regions is hardly possible. The price for a covered TIPS stent in Europe is about €3.000. Therefore, the price of consumables for TIPS implantation may be significantly higher than for shunt surgery, but the lower invasiveness is likely to be reflected in shorter lengths of hospital stay and intensive care treatment. On the other hand, TIPS may require more frequent follow-up and additional pharmacotherapy.

The study has some limitations, especially due to the small number of cases as a consequence of the rarity of HSS in Europe, and the limited follow-up period due to challenging long-term evaluation in this particular patient collective (e.g. patients with refugee status were re-allocated several times and thus lost to follow-up). Moreover, most patients in this study were refugees resulting in a bias in countries of origin, as few endemic countries account for higher numbers of refugees e.g. Eritrea. Because of the special circumstances of the rise in refugee numbers in 2014/2015 we cannot expect rising cases of HSS requiring TIPS insertion in Europe in the near future. Patients in our cohort also differed in terms of comorbidities, age, and possible duration of infection.

Diagnostic procedures and therapeutic algorithms were not aligned between the centers. However, since all centers adopt to current evidence based guidelines, no major differences in indication making and treatment were apparent.

The data presented have been collected retrospectively and no control cohort is available. The study therefore does not allow differentiation between shunt surgery and TIPS in terms of complication rates, long-term survival, and DALYs. These questions will have to be answered by prospective controlled studies.

## Conclusion

TIPS is an effective and safe treatment option in carefully selected HSS patients. The success in terms of reduction of varices and bleeding prophylaxis was excellent. The intervention and associated hemodynamic changes were well tolerated in most cases. If TIPS implantation proves technically unfeasible, surgical treatment remains a useful alternative.

TIPS intervention and shunt surgery require a high level of expertise and equipment. Therefore they do not yet represent a broad treatment option for the majority of endemic regions. However, in endemic countries with advanced healthcare systems, such as Brazil or some regions in Africa and Asia, implementation may be feasible. Larger prospective studies are recommended in such regions to verify our results.
